# Broadband Circularly Polarized Slot Antenna Loaded by a Multiple-Circular-Sector Patch

**DOI:** 10.3390/s18051576

**Published:** 2018-05-15

**Authors:** Son Trinh-Van, Youngoo Yang, Kang-Yoon Lee, Keum Cheol Hwang

**Affiliations:** School of Electronic and Electrical Engineering, Sungkyunkwan University, Suwon 440-746, Korea; jsonbkhn@gmail.com (S.T.-V.); yang09@skku.edu (Y.Y.); klee@skku.edu (K.-Y.L.)

**Keywords:** broad bandwidth, circular polarization, microstrip feed line, multiple-circular-sector configuration, slot antenna

## Abstract

In this paper, a microstrip-fed broadband circularly polarized (CP) slot antenna is presented. CP operation can be attained simply by embedding an S-shaped strip. By loading with a multiple-circular-sector patch, which consists of 12 circular-sector patches with identical central angles of 30° and different radii, the 3 dB axial ratio (AR) bandwidth is significantly broadened. To validate the performance of the proposed antenna, an antenna prototype is fabricated and tested. The fabricated antenna is 54 mm × 54 mm × 0.8 mm in size. The measured −10 dB reflection and 3 dB AR bandwidths are 81.06% (1.68–3.97 GHz) and 70.55% (1.89–3.95 GHz), respectively. Within the 3 dB AR bandwidth, the measured peak gain is 3.81 dBic. Reasonable agreement is also obtained between the measured and simulated results.

## 1. Introduction

Microwave imaging is a promising technique for various applications, such as security, non-destructive testing and evaluation, medical imaging, and through wall imaging. In order to achieve high resolution images, sensitive antennas are used for electric-field mapping of short-time microwave pulses. Due to the capability of mitigating multipath propagation effects and providing better flexibility with regard to the orientation angle between the transmitter and receiver, circularly polarized (CP) antennas are a very desirable feature in microwave imaging sensing systems and communication applications [1,2,3,4,5]. In recent years, with the rapid development of modern wireless communications, the demand for broadband CP antennas has increased considerably. With the well-known advantages of a wide bandwidth and low-cost manufacturing, the printed slot antenna has become a promising candidate for CP antenna designs, where enhanced 3 dB axial ratio bandwidths (ARBWs) are needed. Various broadband CP slot antennas have been developed [6,7,8,9,10,11,12,13,14,15,16]. To realize circular polarization, two orthogonal modes with equal amplitudes and 90° phase difference should be excited. For slot antennas, this can be realized by adding perturbation structures to the feed line and slot. For example, a square slot antenna fed by an asymmetric coplanar waveguide (CPW) was designed to have a 3 dB ARBW of 30.63% by implanting a pair of grounded strips and having an inverted-L tuning stub protrude from the feed line [6]. In another study [7], a circular CPW-fed CP slot antenna with a 3 dB ARBW of 36.01% was realized by having a T-shaped metallic strip protrude from the ground plane towards the slot center. By utilizing a lightening-shaped feed line and a pair of inverted-L grounded strips, a 3 dB ARBW of 48.82% was attained [8]. To improve the CP bandwidth further, CP slot antennas with specially shaped slots were also investigated. For example, a CP slot antenna with a 3 dB ARBW of 28.81% was realized in a design which used a Spidron fractal slot embedded on a Spidron fractal patch [9]. Symmetric slot antennas were also presented and designed in effort to improve the ARBW [10,11,12]. A microstrip-fed ring slot antenna with a pair of grounded hat-shaped patches was introduced [13], reportedly showing a wide 3 dB ARBW of 56.79%. In another study [14], a CP slot antenna design with a 3 dB ARBW of 57.43% was realized by using a multiple-circular-sector slot. In other works [15,16], an S-shaped slot and an L-shaped slot antennas were used, with 3 dB ARBWs of 58.66% and 60.87% attained, respectively.

In this paper, we propose the design of a microstrip-fed broadband CP slot antenna. By embedding an S-shaped strip, which is attached to the signal line, CP radiation is attained [13]. The CP bandwidth is then significantly enhanced by loading a multiple-circular-sector patch inside the slot to serve as a perturbation structure that can generate two orthogonal modes with identical amplitudes and in-phase quadrature components. As a result, the 3 dB ARBW of the proposed antenna is enhanced by as much as 70.55% and is completely enclosed by a −10 dB reflection bandwidth of 81.06%. The measured CP operating bandwidth of the proposed antenna is 1.89–3.95 GHz, covering the operating frequency bands of the following systems: Bluetooth/WLAN (2.4–2.484 GHz), LTE band 1 (2100 MHz), and LTE band 7 (2600 MHz). In addition, it is also important to note that the proposed antenna can be directly scaled in order to work with other frequency bands. The proposed antenna with the key features of a more compact size and a broader 3 dB ARBW can be considered as an ameliorated design of an earlier antenna [14] that also uses the multiple-circular-sector configuration. Details of the antenna design and the experimental results are presented and discussed in the following sections.

## 2. Antenna Design and Parametric Studies

### 2.1. Antenna Design

**Figure 1:**
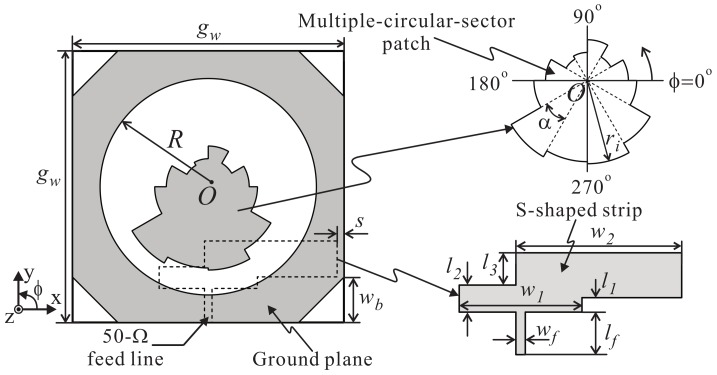
Geometry of the proposed antenna.

Figure 1 illustrates the geometry of the proposed antenna, which consists of a ground plane incorporating a circular slot, a multiple-circular-sector patch, an S-shaped strip, and a 50-Ω microstrip feed line. The multiple-circular-sector patch and the ground plane are mounted on the upper layer of an FR-4 substrate with permittivity of 4.4, a thickness of 0.8 mm, a loss tangent of 0.025, and square dimensions of $g_{w}\times g_{w}$. The circular slot has a radius of *R* and its center is *O*. The four corners of the ground plane are mitered with a width of $w_{b}$. The 50-Ω microstrip feed line with a width of $w_{f}$ and a length of $l_{f}$ and the S-shaped strip are printed on the bottom layer of the substrate. The S-shaped strip is attached to one end of the 50-Ω microstrip feed line. This S-shaped strip consists of three rectangular strips with dimensions of $w_{1}\times l_{1}$, (*w*_1_/2 − *w_f_*/2 + $w_{2}$) × ($l_{2}$ − $l_{1}$), and $w_{2}$ × $l_{3}$. The $l_{2}$ section is symmetrically broadened to have a width of $w_{1}$. Given that $w_{1}$ > $w_{f}$, the feeding structure is referred to as a deformed bent feeding line, which can improve the impedance matching of the proposed antenna. Meanwhile, the $l_{3}$ section is extended toward the right side to have a width of $w_{2}$. This extended section is referred to as a perturbation structure, which can broaden the CP operating band of the proposed antenna [13]. Here, *s* denotes the spacing between the rightmost edge of the S-shaped strip and the right edge of the substrate. The concept of the multiple-circular-sector configuration has been implemented in a CP slot antenna [14] and in an ultra-wideband patch antenna [17]. Recently, this configuration was also used to design a wideband CP dielectric resonator antenna [18]. In the present work, the multiple-circular-sector configuration is investigated and adopted into a slot antenna to realize broadband CP operation. Unlike an earlier antenna [14] in which the multiple-circular-sector configuration was used to form a specially shaped radiating slot, the proposed antenna is designed as a wide circular slot antenna loaded with a multiple-circular-sector patch inside to serve as a perturbation structure to generate two orthogonal modes with identical amplitudes and in-phase quadrature components. As shown in Figure 1, the multiple-circular-sector patch is composed of 12 small circular-sector patches, which have identical central angles of α = 30°, all centered at *O*. These circular sectors have different radii $r_{i}$ (*i* = 1 to 12). All of the dimensional parameters are optimized using the internal *Genetic Algorithm* optimizer of the ANSYS High-Frequency Structure Simulator (HFSS) with an iteration number of 1000, a population of 20, a mutation rate of 0.15, and a single-point crossover scheme. The cost function is defined as:

(1) $$cost=\frac{1}{N}\sum_{n=1}^{N}S_{11}\left(f_{i}\right)+\frac{1}{N}\sum_{n=1}^{N}AR\left(f_{i}\right)$$

where

(2) $$S_{11}\left(f_{i}\right)=\left\{\begin{array}{cc} 0 & ,\;\mathrm{for}\;S_{11}\left(f_{i}\right)\leq -10\;dB\\ S_{11}\left(f_{i}\right)+10 & ,\;\mathrm{for}\;S_{11}\left(f_{i}\right)>-10\;dB \end{array}\right.$$

and

(3) $$AR\left(f_{i}\right)=\left\{\begin{array}{cc} 0 & ,\;\mathrm{for}\;AR(f_{i})\leq 3\;dB\\ AR(f_{i})-3 & ,\;\mathrm{for}\;AR(f_{i})>3\;dB \end{array}\right.$$

with $f_{i}$ = (1.7, 1.8, 1.9, …, 3.9, 4.0) being the set of sampled frequencies of the design frequency band. The optimization goal is set to maximize the overlapping bandwidth (−10 dB reflection bandwidth and 3 dB ARBW). Table 1 summarizes the final optimized dimensional parameters of the proposed antenna.

**Figure 2:**
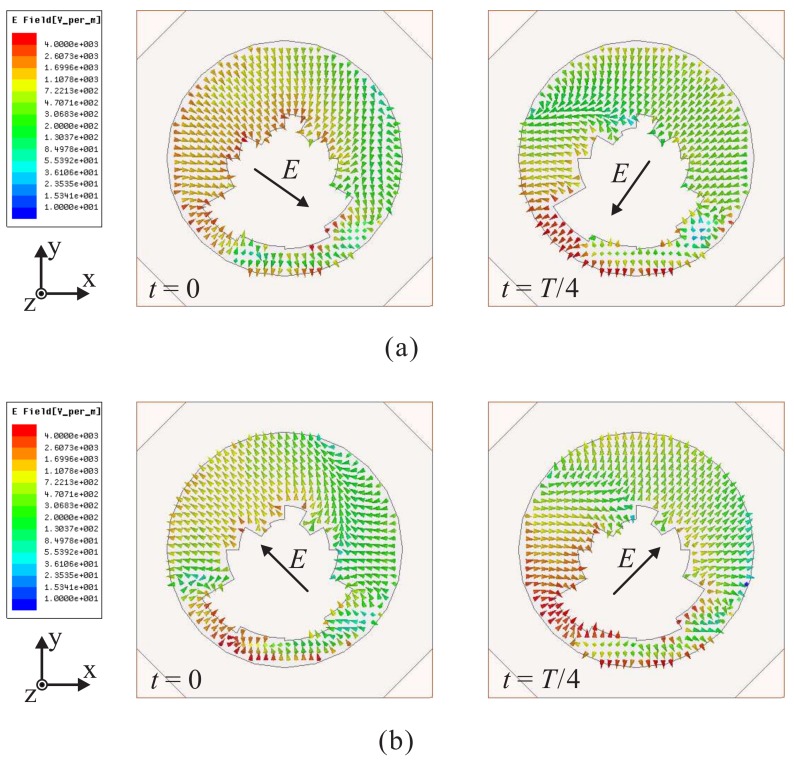
Simulated electric field distributions in the slot with period *T* at: (**a**) 2.1 GHz; (**b**) 3.5 GHz.

To visualize how the CP radiation is realized, Figure 2 displays the time-varying electric field in the slot, which is observed in the +*z*-direction, at *t* = 0 and *T*/4 for 2.1 GHz and 3.5 GHz, respectively. Note that *E* represents the vector sum of the dominant electric field components. As indicated in Figure 2a, at *t* = 0, vector *E* is oriented from the upper left corner to the lower right corner. Meanwhile, at *t* = *T*/4, vector *E* points from the upper right corner to the lower left corner. These vectors are orthogonal and rotate in a clockwise fashion as the time *t* is increased. This indicates that the proposed antenna is able to realize left-handed circular polarization (LHCP) in the +*z*-direction, whereas right-handed circular polarization (RHCP) is generated in the −z-direction. The same circumstances are observed at 3.5 GHz, as shown in Figure 2b.

### 2.2. Parametric Studies

**Figure 3:**
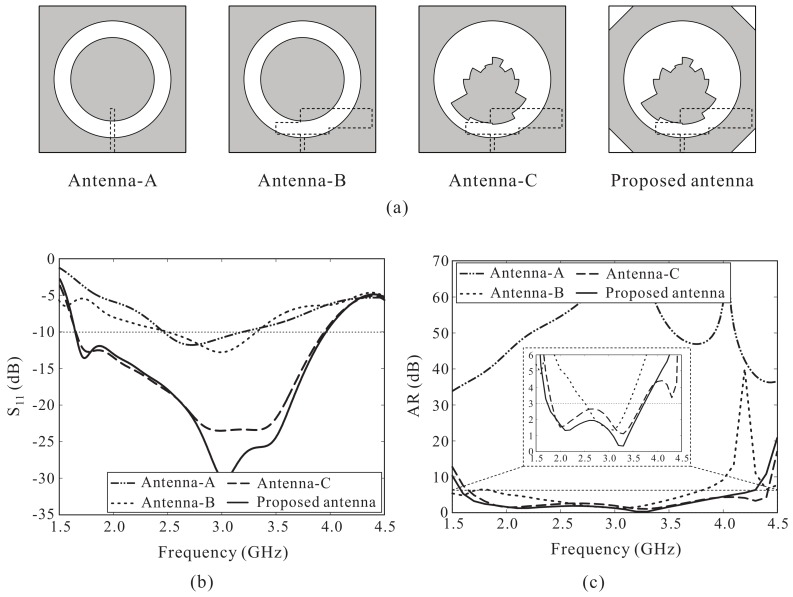
(**a**) Design process of the proposed antenna; (**b**) Simulated reflection coefficient; (**c**) Simulated axial ratio (AR).

With the goal of increasing the CP bandwidth, we modified the structure based on a traditional microstrip-fed slot antenna. Figure 3a illustrates the design process of the proposed antenna. First, Antenna-A is a traditional microstrip-fed slot antenna loaded with a circular patch, and Antenna-B is designed with an S-shaped strip which protrudes from the feeding line. Next, the circular patch of Antenna-B is replaced by a multiple-circular-sector patch to form Antenna-C. Finally, the proposed antenna is designed by mitering four corners of the ground plane of Antenna-C. Simulated results of the reflection coefficients and axial ratios (ARs) of these four antennas are illustrated in Figure 3b,c. These figures clearly show that Antenna-A exhibits linearly polarized characteristics. By embedding the S-shaped strip, Antenna-B can produce a CP band of 2.60–3.35 GHz (25.21%). Meanwhile, Antenna-C loaded with the multiple-circular-sector patch instead of the circular patch can significantly broaden the reflection and AR bandwidths. A wide CP bandwidth of 1.82–3.69 GHz (67.88%) is achieved for Antenna-C, approximately 2.5 times the CP bandwidth of Antenna-B. In addition, it was found that the mitered corners do not have a considerable effect on the reflection coefficient performance but slightly increase the 3 dB ARBW as well as improve the AR levels in the middle frequency range. Therefore, the final design, i.e., the proposed antenna, is based on the antenna with the mitered corners.

**Figure 4:**
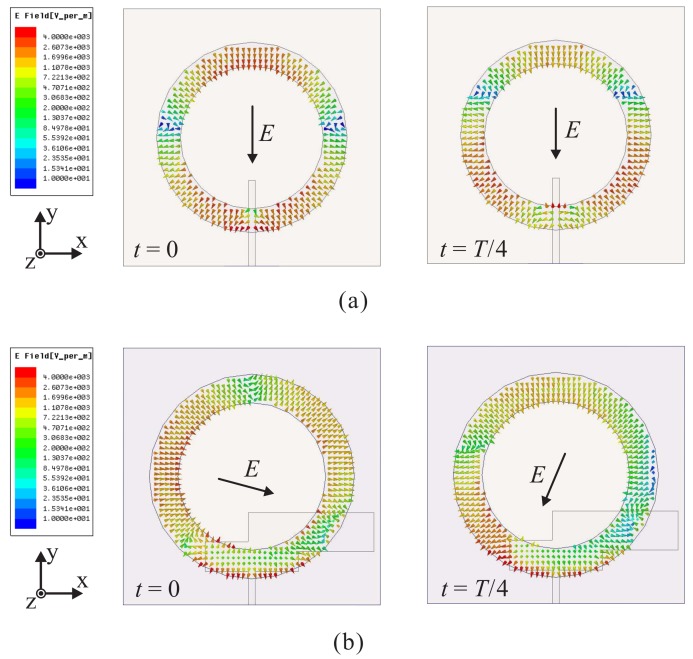
Simulated electric field distribution in the slot with period *T* at 3 GHz for: (**a**) Antenna-A; (**b**) Antenna-B.

Figure 4 shows the time-varying electric field in the slot, as observed from the +*z*-direction, of Antenna-A and Antenna-B at *t* = 0 and *T*/4 for 3 GHz. For Antenna-A, it is found that the electric field direction does not change as the time *t* is increased, as illustrated in Figure 4a. This confirms that Antenna-A is a linearly polarized antenna. Meanwhile, for Antenna-B, the electric field direction changes with an increase in the time *t* (see Figure 4b). The sum vectors of the dominant electric field components at t=0 and t=T/4 are orthogonal and rotate in a clockwise fashion, generating LHCP operation at 3 GHz. In addition, because the mitered corners have a minor effect on the performance of the antenna, the electric field distributions of Antenna-C and the proposed antenna are nearly identical. Therefore, Figure 2 also illustrates the electric field distribution in the slot of Antenna-C. As shown in Figure 2, when loading the multiple-circular-sector patch instead of the circular patch, circular polarization can be achieved at different frequencies, resulting in a broad CP operating bandwidth. This verifies the results shown in Figure 3b.

**Figure 5:**
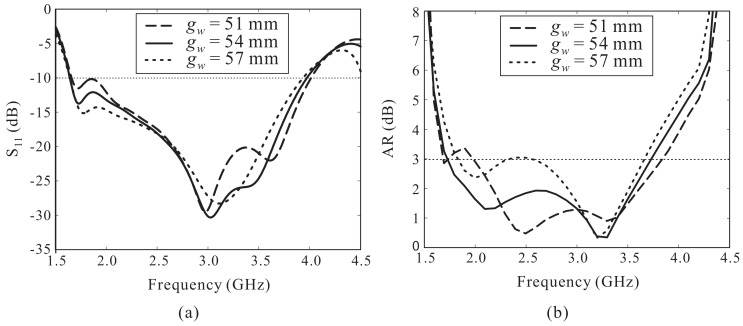
Effect of $g_{w}$ on the antenna performance: (**a**) Reflection coefficient; (**b**) Axial ratio (AR).

Figure 5 shows the effects of the ground plane size $g_{w}$ on the reflection coefficient and AR performance. The reflection coefficient levels at the lower frequencies are improved while the −10 dB reflection bandwidth shows a slight decrease with an increase in the value of $g_{w}$ (see Figure 4a). As shown in Figure 4b, varying $g_{w}$ has considerable effects on the AR performance. When $g_{w}$ = 51 mm, the AR value around 1.9 GHz increases until it exceeds 3 dB. Meanwhile, the CP performance was found to decline at approximately 2.5 GH when $g_{w}$ exceeded 57 mm. Thus, the value of $g_{w}$ is set to 54 mm.

**Figure 6:**
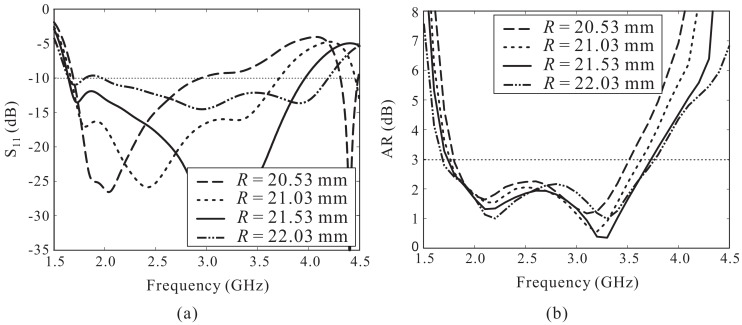
Effect of *R* on the antenna performance: (**a**) Reflection coefficient; (**b**) Axial ratio (AR).

Figure 6 presents the simulated results of the reflection coefficients and ARs when varying the value of *R*. A significant improvement of the −10 dB reflection bandwidth is observed with an increase of *R*, with little influence on the AR performance of the proposed antenna. However, the impedance matching in the lower frequency range is degraded when *R* exceeds 22.03 mm. According to this analysis, the best value of *R* is 21.53 mm, and this value is used here.

## 3. Experimental Verification

**Figure 7:**
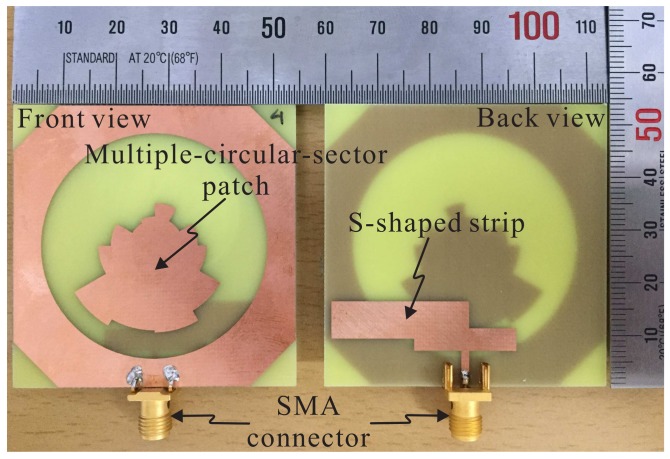
Photograph of the fabricated antenna. SMA: SubMiniature version A.

**Figure 8:**
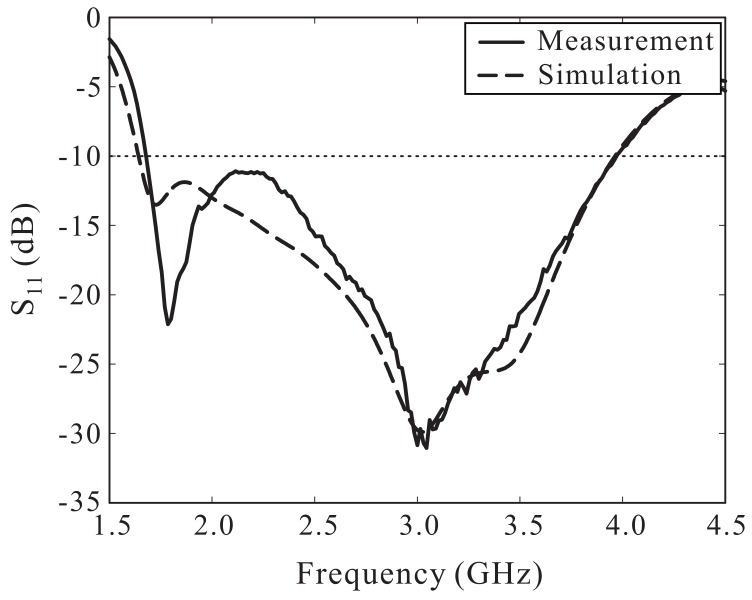
Simulated and measured results of the reflection coefficients.

Based on the optimized dimensional parameters listed in Table 1, an antenna prototype was fabricated for measurement. Figure 7 shows a photograph of the fabricated antenna, with overall dimensions of 54 mm × 54 mm × 0.8 mm. The measurement of the reflection coefficient was done using an Agilent 8510C vector network analyzer and the result was compared to the simulated result, as illustrated in Figure 8. The simulated result is in good agreement with the measured result. A −10 dB reflection bandwidth of 81.06% (1.68–3.97 GHz) was found during the measurement as compared with the bandwidth of 82.86% (1.64–3.96 GHz) determined via the simulation.

**Figure 9:**
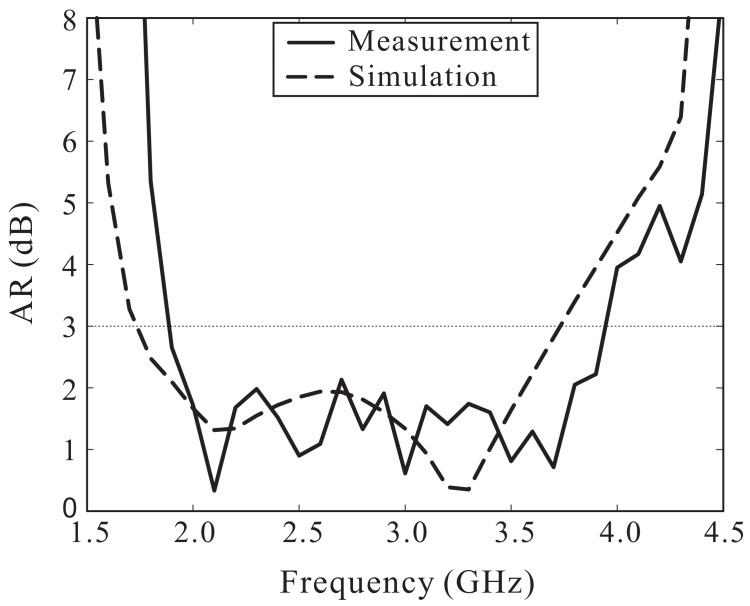
Simulated and measured axial ratios (ARs) versus the frequency.

**Figure 10:**
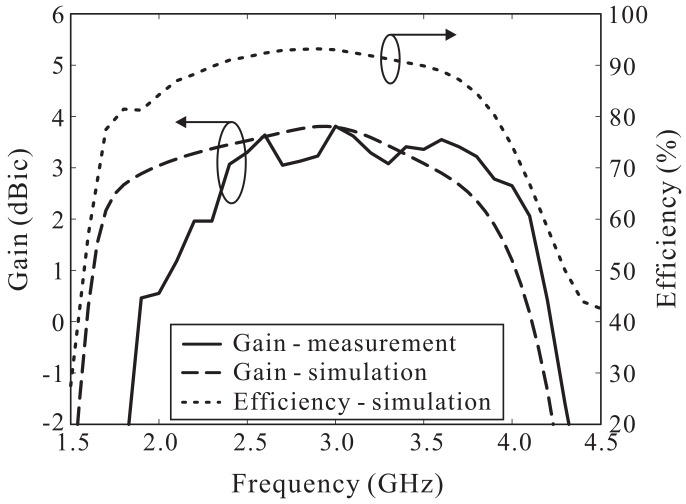
Simulated and measured left-handed circular polarization (LHCP) gains and simulated antenna efficiency.

The simulated and measured ARs versus the frequency are shown in Figure 9. The simulated and measured 3 dB ARBWs in the broadside direction (θ = 0°) were found to be 72.76% (1.74–3.73 GHz) and 70.55% (1.89–3.95 GHz), respectively. Note that the 3 dB ARBW is entirely enclosed by the −10 dB reflection bandwidth. The simulated and measured LHCP gains of the proposed antenna are illustrated in Figure 10. Within the measured 3 dB ARBW, measured LHCP gain varies from 0.46 to 3.81 dBic. Reasonable agreement between the simulation and measurement outcomes was noted, along with some discrepancies caused by fabrication imperfections and/or experimental tolerances. In addition, Figure 10 shows the simulated antenna efficiency, which ranges from 80% to 93% within the 3 dB AR band.

**Figure 11:**
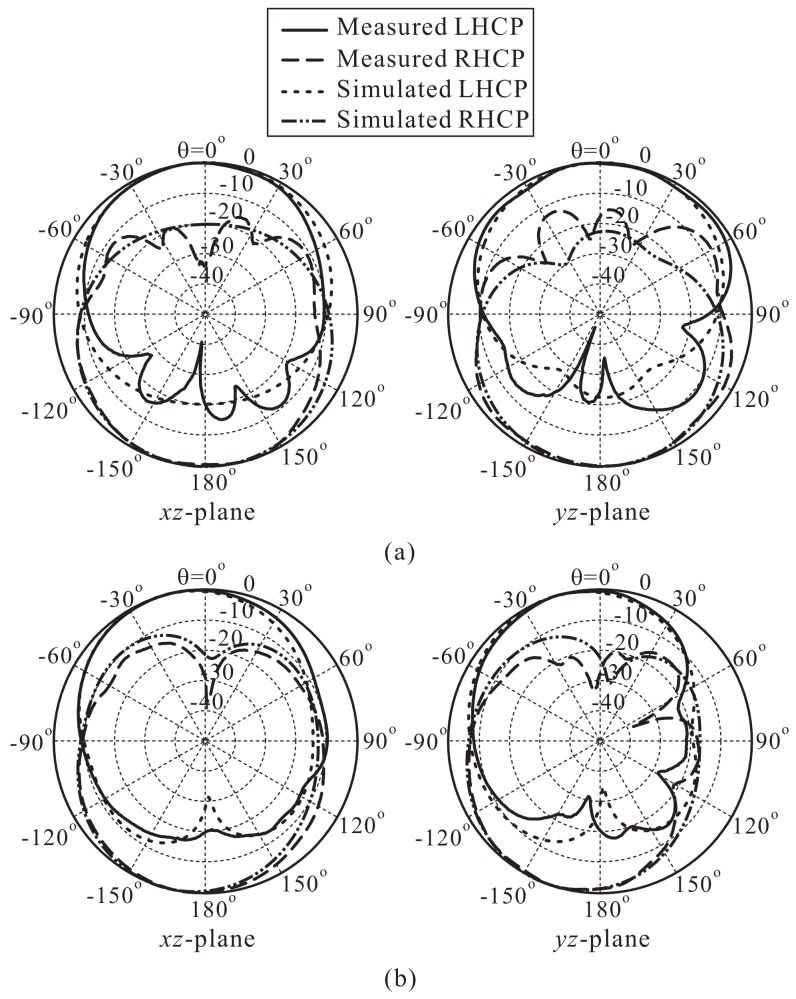
Simulated and measured normalized radiation patterns of the proposed antenna: (**a**) 2.1 GHz; (**b**) 3.5 GHz. RHCP: Right-handed circular polarization.

The results of the simulated and measured normalized radiation patterns on the xz- (ϕ = 0°) and yz- (ϕ = 90°) planes for 2.1 GHz and 3.5 GHz are displayed in Figure 11. It can be observed that the proposed antenna radiates a bidirectional wave with opposite circular polarization. LHCP is realized in the +z-direction, while RHCP is radiated in the −z-direction. In addition, the radiation patterns are asymmetric and somewhat titled, which is mainly caused by the asymmetrical design of the proposed multiple-circular-sector patch. Note that for realizing unidirectional radiation and improving the antenna gain, a metallic reflector can be placed underneath the proposed antenna at a proper distance (approximately one quarter wavelength at the center frequency of the CP band).

**Figure 12:**
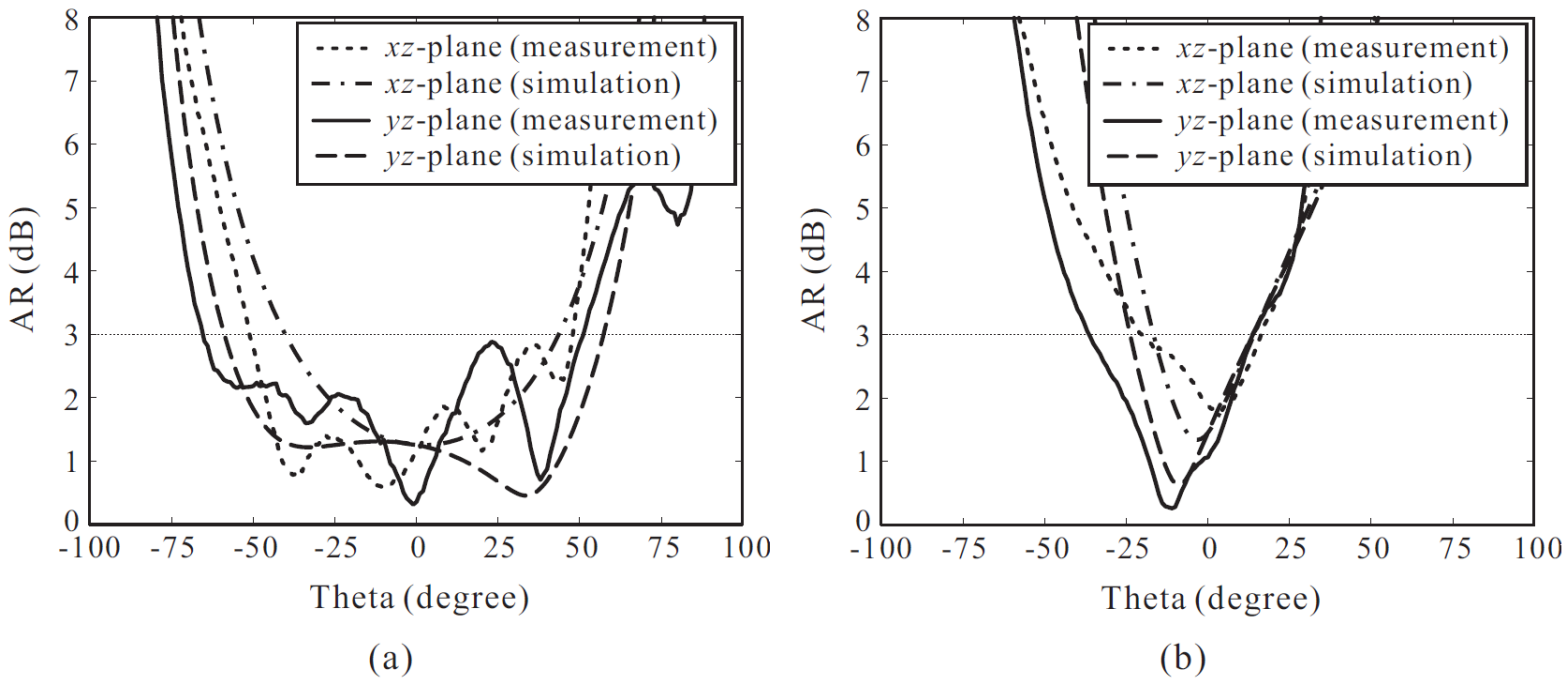
Simulated and measured axial ratios (ARs) of the proposed antenna versus the observation angle: (**a**) 2.1 GHz; (**b**) 3.5 GHz.

Figure 12 illustrates the simulated and measured ARs versus the observation angle of the proposed antenna at two frequencies of 2.1 GHz and 3.5 GHz. It is observed that the 3 dB AR beamwidth becomes narrower with an increase in the frequency. This occurs due to the higher cross-polar levels that can be found in radiation patterns at higher frequencies. At 2.1 GHz, the measured 3 dB AR beamwidths are 100° and 116° on the xz- and yz-planes, respectively. In contrast, the measured 3 dB AR beamwidths at 3.5 GHz are only 39° on the xz-plane and 50° on the yz-plane. In addition, reasonable agreement between the simulation and measurement results is achieved.

Table 2 presents the results of a comparison of the antenna size, the −10 dB reflection bandwidth, the 3 dB ARBW, and the peak gain of the proposed antenna and several CP slot antennas introduced in previous studies [6,7,8,13,15,16], which are bidirectional CP slot antennas. In this table, $\lambda_{c}$ is the wavelength corresponding to the center frequency $f_{c}$ of the 3 dB ARBW. It is evident that the proposed antenna has a broader 3 dB ARBW than all of the previous antennas. In addition, the proposed antenna exhibits an acceptable gain for a common slot antenna. It can also be seen that the antenna size of 0.53 × 0.53 $\lambda_{c}^{2}$ is more compact than those in the earlier works [7,15,16].

## 4. Conclusions

A microstrip-fed broadband CP slot antenna was proposed using the concept of a multiple-circular-sector configuration. The proposed antenna was fabricated using an inexpensive FR-4 substrate and was measured. Reasonable agreement was achieved between the measured and simulated results. The measurement results exhibit a broad CP bandwidth of 70.55% (1.89–3.95 GHz), which is entirely enclosed by an impedance bandwidth (|$S_{11}$| ≤ −10 dB) of 81.06% (1.68–3.97 GHz), and a peak gain of 3.81 dBic. The CP operating frequency range of the proposed antenna covers the operating bands of several systems, such as Bluetooth/WLAN (2.4–2.484 GHz), LTE band 1 (2100 MHz), and LTE band 7 (2600 MHz). In addition, the antenna has a compact size of 0.53 × 0.53 $\lambda_{c}^{2}$. This makes it feasible for use as a low-cost broadband CP antenna for various wireless communication and wireless power transfer applications.

## Figures and Tables

**Table 1:** Optimized Dimensional Parameters of the Proposed Antenna.

Parameter	Value	Parameter	Value
$r_{1}$	8.15 mm	*R*	21.53 mm
$r_{2}$	5.38 mm	$g_{w}$	54 mm
$r_{3}$	8.10 mm	$w_{b}$	9 mm
$r_{4}$	5.58 mm	$w_{f}$	1.50 mm
$r_{5}$	4.91 mm	$l_{f}$	6.77 mm
$r_{6}$	8.34 mm	*s*	1.35 mm
$r_{7}$	10.66 mm	$w_{1}$	19.55 mm
$r_{8}$	17.53 mm	$w_{2}$	26.40 mm
$r_{9}$	16.16 mm	$l_{1}$	2.30 mm
$r_{10}$	16.57 mm	$l_{2}$	4.30 mm
$r_{11}$	14.41 mm	$l_{3}$	5.14 mm
$r_{12}$	9.80 mm	α	30°

**Table 2:** Comparison of the proposed antenna and those in previous studies. ARBW: Axial ratio bandwidth.

Design	$f_{c}$ (GHz)	−10 dB Reflection Bandwidth (%)	3 dB ARBW (%)	Antenna Area ($\lambda_{c}^{2}$)	Peak Gain (dBic)
[6]	2.22	37.61	30.63	0.44 × 0.44	4.1
[7]	2.30	50.12	36.01	0.71 × 0.63	3.9
[8]	2.75	51.36	48.82	0.55 × 0.55	4.2
[13]	3.04	78.31	56.79	0.46 × 0.46	4.1
[15]	4.03	104.04	58.56	0.73 × 0.73	3.55
[16]	4.6	109.58	60.87	0.92 × 0.92	2.2
This work	2.73	81.06	70.55	0.53 × 0.53	3.81
